# Combining Repetitive Transcranial Magnetic Stimulation and Video Game-Based Training to Improve Dexterity in Parkinson's Disease: Study Protocol of a Randomized Controlled Trial

**DOI:** 10.3389/fresc.2021.777981

**Published:** 2021-11-23

**Authors:** Manuela Pastore-Wapp, Dirk Lehnick, Tobias Nef, Stephan Bohlhalter, Tim Vanbellingen

**Affiliations:** ^1^Neurocenter, Luzerner Kantonsspital, Lucerne, Switzerland; ^2^ARTORG Center for Biomedical Engineering Research, Gerontechnology and Rehabilitation Group, University of Bern, Bern, Switzerland; ^3^Biostatistics and Methodology, Clinical Trials Unit Central Switzerland, Lucerne, Switzerland; ^4^Department of Health Sciences and Medicine, University of Lucerne, Lucerne, Switzerland; ^5^University of Zurich, Zurich, Switzerland

**Keywords:** Parkinson's disease, transcranial magnetic stimulation, dexterity, video game-based training, RCT - randomized controlled trial

## Abstract

**Introduction:** Patients with Parkinson's disease (PD) often exhibit difficulties with dexterity during the performance of activities of daily living (ADL) due to dysfunctional supplementary motor area (SMA). The aim of this clinical trial protocol work is to describe how the effectiveness of a combined repetitive transcranial magnetic stimulation (rTMS) over SMA and video-game-based skill training (VBT) in PD will be evaluated. The short and long-term benefits are assessed.

**Methods and analysis:** A single-blind (patients) stratified (based on Hoehn & Yahr) parallel randomized sham-controlled rTMS-VBT study with a baseline and two follow-up measurements (3 and 12 weeks) is being conducted. These measurements include the dexterity questionnaire 24 (DextQ-24) as a primary outcome, and nine hole peg test and coin rotation task as main secondary dexterity outcomes. Further secondary outcomes will be the subscale II of the movement disorders society unified PD rating scale (MDS-UPDRS) to assess improvements on overall ADL and the Parkinson's Disease Questionnaire-39 to assess quality of life. Thirty-six outpatients (from one neurorehabilitation center) with PD (diagnosis based on brain bank criteria) will be recruited who report difficulties with dexterity in performing ADL. All PD patients will receive a 45-min VBT three times a week for 3 weeks. The PD patients randomized in the experimental group will receive VBT preceded by real rTMS, being intermittent theta burst (iTBS) stimulation sessions. The PD patients randomized to the control group receive a VBT with sham rTMS.

**Discussion:** The study will provide evidence to determine whether a combined iTBS and VBT skill intervention is more effective than a VBT intervention alone to improve dexterity in PD.

**Ethics and dissemination:** The study was approved by the Ethics Committee for Northwest and Central Switzerland (EKNZ), Switzerland 2019–00433. The study will be conducted in accordance with the Helsinki Declaration and the Guidelines of Good Clinical Practice. Informed consent will be signed prior to subject enrolment. Dissemination will include submission to international peer-reviewed professional journals and presentation at international congresses.

The study protocol has been registered in the clinicaltrials.gov registry with the identification code: NCT04699149.

## Introduction

Patients with Parkinson's disease (PD) often face dexterity-related difficulties, both in performing basic (grooming, buttoning a shirt) and instrumental activities of daily living (ADL), such as cooking a meal, organizing pills in pill holders, and writing (1–4). These difficulties may be present even in early stages of the disease. They further increase the burden of disease and reduce quality of life (QoL) (2). Dopaminergic therapy only slightly improves impaired dexterity (5). Therefore, complementary treatments are needed to reduce its impact on ADL. Previous studies have shown short-term effects (immediately after training) of a particular hand training, either supervised (6) or unsupervised (7). However, no sustainable long-term effects were shown so far. A reason may be that important aspects for an optimized motor learning (8) were not sufficiently targeted, such as the variability of the load, the feedback of the performance over a longer period of time or the level of difficulty.

Video-game-based training (VBT) has been rapidly developing in PD neurorehabilitation (9). VBT is attractive and challenging, and therefore potentially suitable to motivate PD patients over time (10). Other benefits of VBT include the ability to adjust the difficulty of the exercise and to provide online visual and/or verbal feedback during the training. In terms of dexterity, several studies have now shown that VBT was feasible and improved dexterity in PD patients in the short term (immediately after the intervention) (11–14). However, long-term effects of VBT have not yet been assessed.

To further improve training effects in PD, a combination of behavioral interventions with neuromodulation techniques such as non-invasive repetitive transcranial magnetic stimulation (rTMS) has been proposed (15–18). The rationale behind a combined rTMS and behavioral training is that by applying rTMS before training, the brain may reach an optimal state of learning thereby facilitating subsequent training effects (19). The principle of activity dependent neuroplasticity (20) also suggests that combinations of behavioral training with rTMS might have promise in facilitating long term effects (21). We use herein an intermittent theta-burst protocol (iTBS), a type of rTMS, which is expected to produce behavioral effects not only outlasting the single administration (short term), but also retained after multiple applications (long term) (22–24). iTBS has been shown to be facilitatory in nature by increasing cortical excitability (25–27). When applied over primary motor cortex iTBS may enhance either sensorimotor integration (28) or mood (29) in PD patients. Similarly, if supplementary motor area (SMA) was the target region, beneficial effects on overall motor symptoms were shown by using rTMS (24, 30). The SMA also plays a key role in the generation of self-initiated, multisegmental, complex, voluntary finger movements (31, 32). In PD however, SMA activity can be reduced due to decreased positive efferent feedback arising from the basal ganglia–thalamocortical motor loop (33). Consequently, PD patients may show altered activation patterns in the SMA and less cortico–cortical excitability. Indeed, it was shown that a diminished resting state perfusion in the left SMA in PD explained poor dexterous performance, which was measured by a coin rotation (CR) task (34). These findings further attributed to the role of the SMA controlling for fine finger movements. Interestingly, one previous pilot rTMS study in PD already demonstrated a short-term improvement on handwriting, which requires good dexterous function, when patients received one session of facilitatory rTMS over the left SMA (35).

The aim of the present clinical trial protocol paper is to describe how the effectiveness of a combined iTBS-VBT skill 3-week intervention in PD will be evaluated. The short and long-term benefits of this training program will be evaluated. We predict that the use of iTBS before VBT can further strengthen the training effects and possibly also achieve sustainable long-term effects. Therefore, we expect significantly improved dexterity in both the short and long-term, which leads to improved ADL and QoL in patients with PD.

## Methods and Analysis

### Trial Design

**Figure 1:**
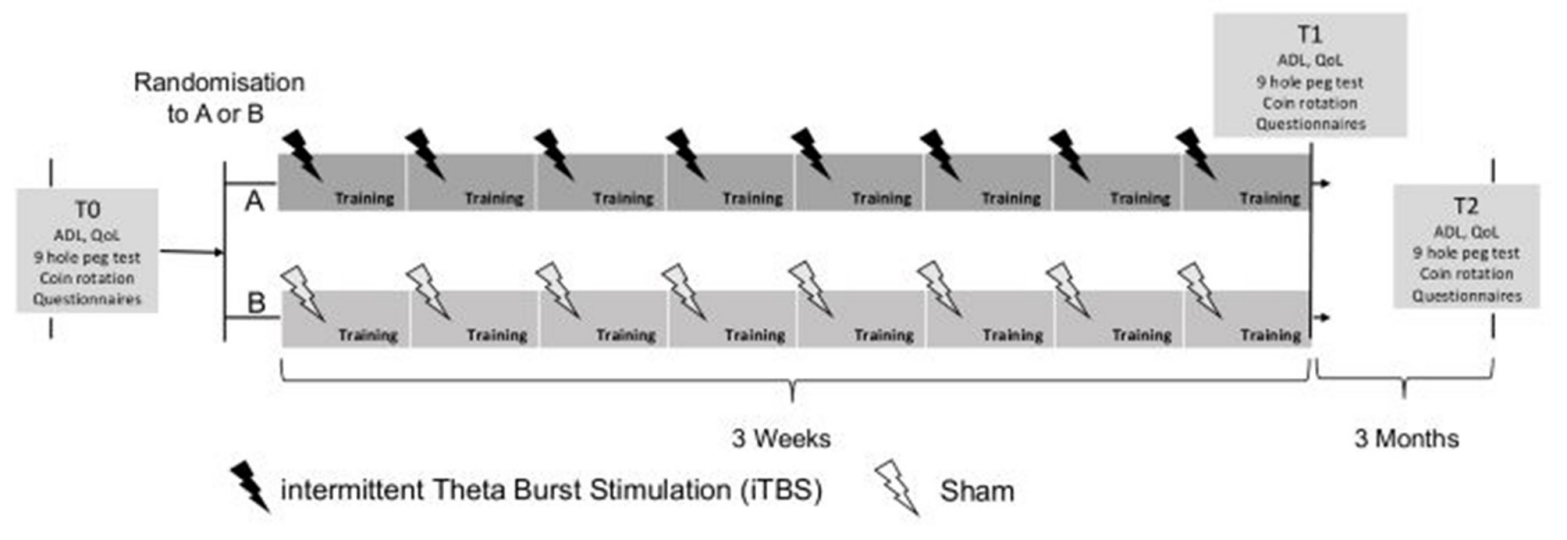
Design of study: T0 baseline assessment, T1 end of training, T2 follow-up after 3 months. ADL, activities of daily living; QoL, quality of life.

A single-blind (patients) parallel RCT with a stratified random intervention distribution is carried out. The random sequence generation using a computer software program is stratified, according to the Hoehn & Yahr (H&Y) scale, Level 1 = H&Y I to Level 4 = H&Y 4, (36) at T0. After stratification, a simple randomization (1–1) occurs within each stratum level (Level 1 to 4). Randomization and treatment allocation is concealed within SecuTrial, which is a GCP-compliant electronic data base system (https://www.secutrial.com/en/), managed externally by the Clinical Trial Unit (CTU) Schweizer Paraplegiker Zentrum (SPZ) Nottwil. After the baseline assessment (hereinafter referred to as T0), all PD patients receive 45-min manual dexterity intervention three times a week for a period of 3 weeks. The PD patients randomized in the experimental group will receive VBT each time, preceded by true iTBS stimulation. The PD patients randomized to the control group receive a VBT with a preceding sham TMS each time (see Figure 1). Patients will be blinded to the rTMS protocol (sham vs. real). This means that patients will not be informed throughout the whole trial whether they received real or sham rTMS stimulation. Follow-up measurements (T1 and T2) are carried out after a period of 3 weeks and 3 months. All data is collected within SecuTrial. The study will be performed according to the CONSORT (Consolidated Standards of Reporting Trials) statement, http://www.consort-statement.org/.

### Participants

The patients are recruited by two investigators (TV, SB), not involved in the assessment and treatment procedures, at the Neurocenter, Lucerne Cantonal Hospital, Switzerland. Inclusion criteria are confirmed PD according to the brain back criteria (37); H&Y I to IV (36); age 50–90 years old; written and signed informed consent and experiencing dexterous difficulties in performing ADL. Exclusion criteria are significant medical, psychiatric comorbidity including dementia as defined by Montreal Cognitive Assessment (MOCA <21) (38); inability to understand the scope of the study and to follow the study procedures according to the protocol, e.g., filling out questionnaires and participating in another intervention study. Exclusion criteria for TMS use are current pregnancy, personal history of epilepsy or seizures, and any psychiatric, neurological, or medical history other than PD. All data related to the study will be collected at the outpatient neurorehabilitation center, Lucerne Cantonal Hospital.

### Sample Size Calculation

The significance level alpha is defined as 0.05 (two-tailed) for detecting a mean difference between groups on the primary outcome being the Dexterity Questionnaire 24 (DextQ-24) (4) in favor of the real iTBS-VBT dexterity group. Based on previously observed data one may assume a within-group standard deviation of up to 10 points. The study is designed to detect a mean difference of 10 or more points, which exceeds the MDC95 of 8 points found in our validation study (4). A total sample size of 30 evaluable subjects, 15 per group, is required to detect a mean difference of an at least 1.1-fold within-group standard deviation with a target power of at least 80%. Considering a maximal drop-out rate of 15%, we aim to recruit 36 patients in total.

In any case, patients who do not complete the training sessions and/or even decide to drop out of the study for whatever reason will be encouraged to continue to participate in the scheduled assessments for at least T1 (and/or a time point prior to the individual end of the study) and ideally to provide a postbaseline assessment for at least the primary endpoint.

### Material

At T0 handedness (39), disease duration, medication dosage intake per day, the Montreal Cognitive Assessment test [MoCA, (40)] are assessed. The MoCA is divided into visuo-spatial abilities, short-term memory, executive function, attention and working memory, language and phonemic fluency, and orientation.

Parkinsonian motor symptoms are assessed by the Movement Disorders Society unified Parkinson's Disease Rating Scale (MDS-UPDRS) subscale III (41) at T0, T1, and T2. Severity of the upper limb motor deficits is measured with the items 3.3 to 3.6 and 3.15 to 3.18 of the MDS-UPDRS subscale III.

### Primary Endpoint

The DextQ-24 is assessed at T0, T1, and T2, which is a standardized patient self-rated outcome measure for evaluating dexterity related ADL in PD (4). This questionnaire contains 24 questions, which are divided into five subgroups (“washing/grooming;” “dressing;” “meals and kitchen;” “everyday tasks;” “TV/CD/DVD”). For each question, patients must state whether they have no problems (1 point), minor problems (2 points), major problems (3 points), or need aid (4 points) to perform the task. Points are added in each subgroup and summed to a total score. Score ranges from a minimum of 24 to a maximum of 96 points.

### Secondary Endpoints

The Nine Hole Peg test (9-HPT) and the coin rotation (CR) task are used to explore hand and finger function at T0, T1, and T2. The 9-HPT is a standardized, well established, and reliable measure of hand performance in patients with PD (42, 43). The patients have to take nine pegs one by one into the holes on a board and then move them back to the container. The CR task (44) measures fine coordinated finger movements and has proven to be a suitable and valid dexterity test in patients with PD (5, 45). The CR task requires the patient to rotate a 20 swiss cent through consecutive 180° half turns, as rapidly as possible for 10 rotations. Time to complete the 9-HPT and the CR tasks are recorded, by an experienced non-blinded outcome assessor (MP-W), twice for each hand separately.

The subscale II of the MDS-UPDRS (41), containing 13 items, and each of them scored on a 0–4 rating scale (0 = normal; 1 = slight problem, 2 = mild problem, 3 = moderate problem, 4 = severe problem), is used to assess improvements on overall ADL. The scale is designed to be a self-administered questionnaire, but can be reviewed by the outcome assessor to ensure completeness and clarity.

To assess QoL (at T0, T1, and T2), we used the Parkinson's disease questionnaire-39 (PDQ-39) (46). This patient self-rated questionnaire consists of 39 questions, which are divided into eight subscales (mobility, ADL, emotional well-being, stigma, social support, cognition, communication, and bodily discomfort). The total score is given by the sum of all items and is then transformed in a range from 0 to 100. A lower value corresponds to a better perception of subject's QoL.

### Stimulation Protocol

iTBS is applied using a MagPro R30 stimulator (Medtronic Functional Diagnostics, Skovlunde, Denmark), connected to a figure-of-eight coil (Magnetic Coil Transducer MC-B70, Medtronic) with an outer radius of 50 mm or to a similar looking sham coil (Magnetic Coil Transducer MC-P-B70, Medtronic). An iTBS protocol is used as done in our previous rTMS study (4). For the iTBS protocol a theta burst of three pulses with a 20-ms interval repeated as a train of 10 bursts with a repetition rate of 5 Hz is used. Trains are repeated 20 times with an interval of 8-s. During stimulation, the examiner places the figure-eight coil over the left or right SMA, depending on the side of the more severe dexterity problems. The position for SMA stimulation is determined in each patient as follows. First, the optimal position for activation of the right abductor hallucis muscle will be determined by moving the coil in 1 cm steps along the sagittal midline around scalp vertex (Cz) with the handle pointing to the right. The active motor threshold (AMT) for this muscle is then determined. Next, stimuli at 120% AMT are given, moving the coil anteriorly along the sagittal mid-line in 1 cm steps. The SMA is defined as being 1 cm anterior to the last site from which motor evoked potentials (MEPs) can be evoked during contraction (47). Following these criteria, the position for SMA stimulation is expected to be 3 cm anterior from the optimal position for activation of the abductor hallucis muscle in most patients. Sham stimulation is applied by the same iTBS protocol, however a sham coil is used. The patients are asked to keep their eyes closed during stimulation. Immediately after stimulation the PD patients start with the VBT.

### VBT Intervention

**Figure 2:**
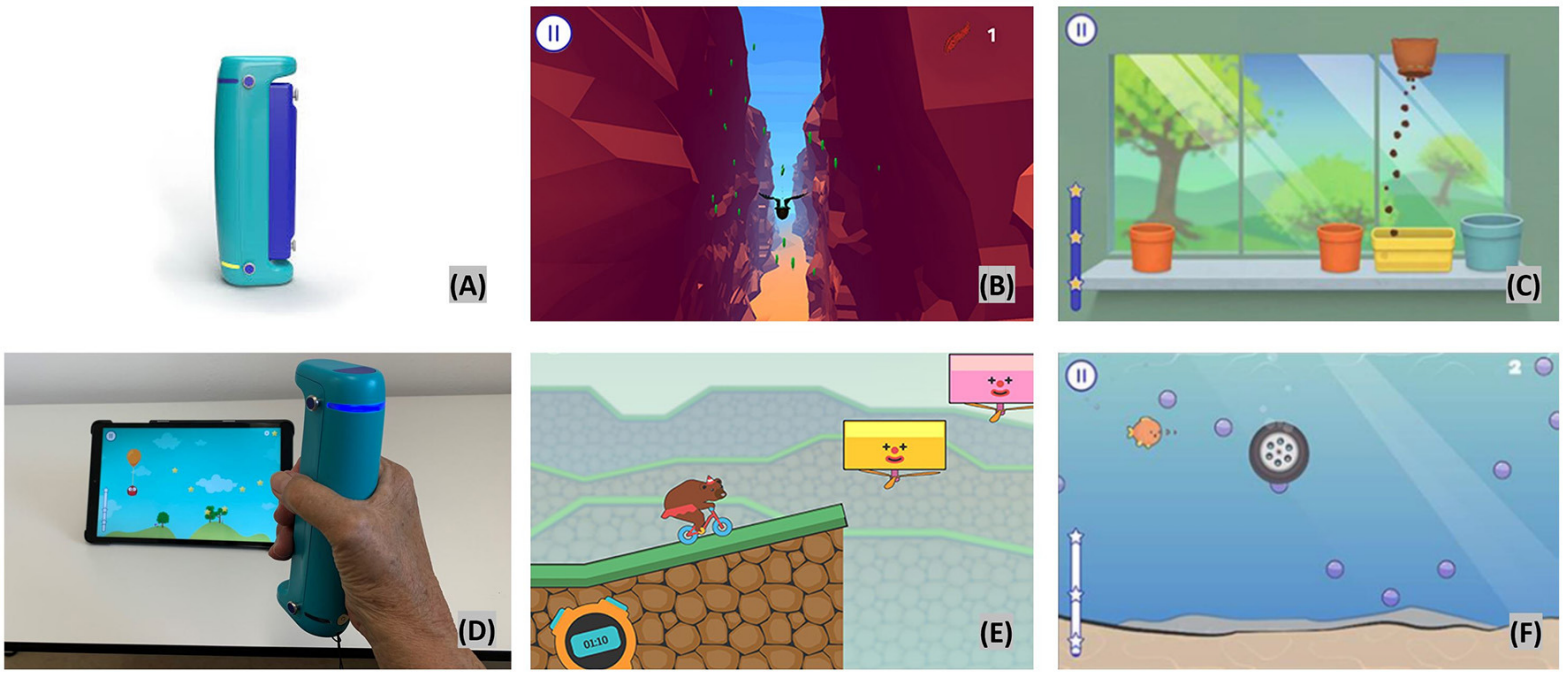
Video based training with GripAble. Top left **(A)** GripAble device; top middle **(B)** Plume; top right **(C)** Windowsill; bottom left **(D)** patient playing Balloon Buddies; bottom middle **(E)** Circus Escape; bottom right **(F)** Pufferfish.

The VBT intervention takes place at the neurorehabilitation outpatient center, Lucerne Cantonal Hospital. The interactive training program contains the use of two different devices. Each device is used for about 15 min. The first interactive device is the GripAble (https://gripable.co/) (Figure 2A), which is a new wireless device allowing the training of upper-arm and hand movements during wrist extension and flexion, pronation and supination, wrist radial and ulnar deviation, and also hand and pinch grip-force (48). To be able to manipulate objects well-one needs good hand/finger pinch grip and release (49, 50). The device is able to capture fine hand/finger movements. It can be connected (by bluetooth) with a tablet on which a GripAble app including different therapy games is installed. So far, nine games are available, of which five were chosen to be used for the present trial, since each of these five games focus on different hand/finger movements. Finger movements (pinch grip) can be done by adding a pinch pin.

#### Plume

In this activity, the user is controlling the bird as it flies along a course using wrist flexion to move downward and wrist extension to move upward. The patient has to collect as many feathers as possible and to avoid different obstacles. If the bird flies in an obstacle the patients lose points. High level of visual perception and concentration is required. The time taken increases as the user works through the levels (Figure 2B top middle).

#### Windowsill

This activity requires wrist pronation, supination, and grip release. This activity presents pots in different places on a windowsill. A bag of soil is then moved left to right (using pronation/supination) until it is placed directly above one of the pots. When still, the soil can be released to fill the pot by gripping. Once the pot is full, a seed can be placed into the pot using the same control. This is followed by a watering can which needs to be poured until the flower appears. As the levels progress, more pots appear and are at a wider spacing (Figure 2C top right).

#### Balloon Buddies

The focus is on controlled grip (requiring hand/finger strength and endurance) and release. The patient controls an owl which is suspended from the balloon. The owl needs to collect all the stars to gain points. Squeezing GripAble inflates the balloon to make the owl go up the screen. Releasing GripAble brings the owl down the screen. The levels give increased time and complexity of the required control. The emphasis is on smooth transition between grip and release. The placement and the smoothness of the curve of stars alters through the levels to give gradual increase in demand (see Figure 2D bottom left).

#### Circus Escape

This activity targets controlled and fast reaction in grip and release. The goal is for the patients to power the bear on the cycle along the course without falling off the cliff. The patient will need to squeeze with more intensity and harder as the cycle moves up hills. The levels increase in complexity and length as they progress. There is a natural break within each level as the user waits for the hazards to reach the point of safe movement (Figure 2E bottom middle).

#### Pufferfish

This activity requires wrist ulnar and radial hand deviation, optional grip and release. The patient is controlling the fish to move up and down the screen by moving GripAble through wrist radial and ulnar deviation. The fish needs to catch the bubbles. In Level 3 onwards, hazards appear which need to be avoided or can be blown away by squeezing. The fish will not be affected by swimming over the sandy area at the base of the screen (Figure 2F bottom right).

**Figure 3:**
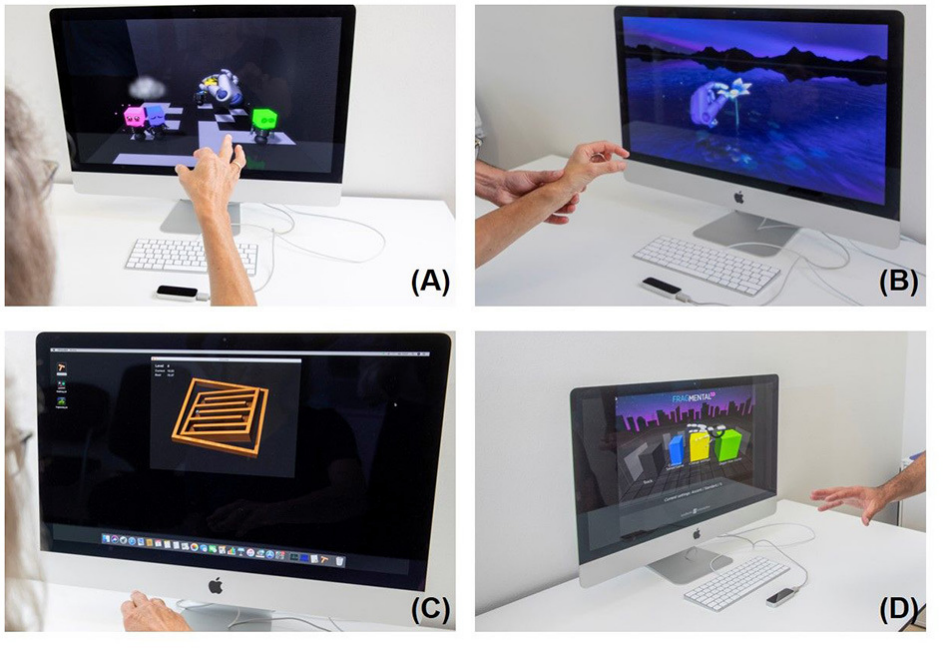
Leap Motion training, Top left **(A)** Blocks; top right **(B)** flower; bottom left **(C)** Tilt Your Ball; bottom right **(D)** Fragmented 3D.

The second device is the Leap Motion™ Controller, LMC™ (https://www.leapmotion.com/) which is an optoelectronic commercially available device suitable for hand gesture-controlled user interfaces allowing human–computer interaction. It tracks hand and finger movements by modelling all physiological hand and finger joints within a virtual reality (VR) environment (51, 52). The patients see their hand in real time on the screen (Figure 3).

Four games were downloaded from the manufacturer's Web site (Leap Motion Gallery: Blocks, Flowers, Tilt Your Ball and Fragmental 3D (see also https://gallery.leapmotion.com/), which were partly evaluated in our previous pilot study (14). In short, in the first game, *Tilt Your Ball* (Figure 3A top left), the screen shows a room filled with four colored blocks. There are also four bodies. The aim is to use the fingers to connect the colored blocks to the body (pinch grip). The second game shows a flower (Figure 3B top right) made of steel with seven pedals. The aim is to remove the pedals one by one with the fingers. In *Tilt Your Ball* (Figure 3C bottom left), the patient has to navigate a small metal ball through different levels by tilting his hand. The successive levels become more and more difficult. *Fragmented 3D* (Figure 3D bottom right) is played by moving, rotating, and dropping blocks to form rows across a 3D grid. The goal is to build rows across the grid to erase lines, make combos and thus score points. The games Blocks, Flower, Fragmented 3D specifically requires fine coordinated movements. Tilt Your Ball requires fine combined wrist flexion extension, forearm prosupination movements, which are all needed for optimal object manipulation.

### Statistical Analysis

Descriptive statistics are used to present baseline characteristics and results of outcome measurements. In order to investigate the treatment effect for the primary endpoint, a hierarchical testing strategy is applied. For DextQ-24 at T1, an ANCOVA model is used with a fixed effect for treatment and the baseline (T0) value of DextQ-24 as a covariate. If a treatment effect at T1 could have been shown (null hypothesis rejected), the same approach will be performed for DextQ-24 at T2 in order to see whether the treatment effect is sustainable. An estimate for the treatment effect will be derived from the model together with a corresponding 95% confidence interval. As a postestimation procedure effect size measures such as (partial) eta square with corresponding 95% intervals will be provided. In an exploratory manner, the secondary endpoints are tested utilizing the same methodology. As a sensitivity analysis, e.g., to explore the robustness of the results in case of missing values, the primary and secondary endpoints are analyzed using linear mixed effects models with fixed effects for treatment, time point, interaction of treatment and time point, baseline value as a covariate and with subject as a random effect. Estimates for the treatment effects at T1 and T2 with corresponding 95% confidence intervals can be obtained as contrasts derived from these models. Further supportive analyses to check the robustness of the treatment effect estimators can be performed by adjusting the model from the primary analysis for the stratifying variable, namely the H&Y scale.

According to the treatment policy strategy (Intention to treat (ITT) principle) every randomized PD patient, including the drop-outs, is included for final evaluation. For all analyses the level of significance is set to alpha = 0.05 (two-tailed). Statistical analyses are performed using Stata (Version 16.1 or later, StataCorp, College Station, Texas, USA).

## Discussion

Dexterity related difficulties during the performance of several ADL are frequently reported in PD, leading to reduced QoL (1–3, 7). Specific dexterity trainings have shown short, (6, 12, 14) but no long-term effects (7). The present study choses an innovative multimodal therapeutical approach, not done before, by using a VBT intervention using two types of devices (GripAble and LMC™), each having its own focus. The GripAble games chosen herein, focus on training train hand/finger pinch grip and release, and different hand movements, which are all key elements for good manipulation of objects. The LMC™ training focus more on independent finger movements. To further boost the effects on dexterity the VBT training is combined with an facilitatory iTBS over SMA, a cortical region being involved in fine motor control (34, 35).

The present proof-of-concept RCT aims at combining, for the first time, VBT and iTBS targeting SMA. The VBT used herein is an attractive new way to train dexterity in PD (9). The games, delivered by using GripAble and LMC™ in this RCT, provide direct feedback, are fun, motivating, and incorporate several levels of difficulty, all aspects which are important to trigger motor learning in PD (8). By combining this attractive training with iTBS we expect to achieve sustainable long-term benefits in dexterity-related ADL also leading to improved QoL in patients with PD. The reason for choosing iTBS is its shorter application time, therefore clinically being more applicable, and lower stimulation intensities compared with conventional rTMS paradigms, inducing more longer-lasting neural effects (23). Currently, TBS seems to be one of the most powerful neuromodulatory stimulation protocols currently available (23).

The total dosage of the present study is 405 min of combined iTBS-VBT training (45 min, three times a week, 3 weeks), representing a short but intensive training. Previous studies using a similar amount of rTMS sessions, already suggested longer lasting behavorial effects, even up to 3 months (53, 54). We assume that a 3-week combined iTBS-VBT training will be enough to achieve both short as well as long-term effects. Furthermore, due to the short training period, it has the potential to be easily implemented in the daily routine of a neurorehabilitation center and at home.

Some limitations have to be mentioned which might occur during the trial. These could be related to the technical devices used in the VBT. The developers of GripAble (see for acknowledgments) are continuously doing efforts to upgrade their system, also by developing new games, potentially interesting for the present trial. However, to avoid contamination of training effects, we will not implement these new games. The long-term follow-up (12 weeks after training) might be a challenge for some PD patients, and may lead to some drop-outs. However, we expect no drop-out rate exceeding 10%, as shown in our previous RCT with a similar design (7).

In summary, the present project aims to investigate, for the first time, the effectiveness of a combined iTBS-VBT 3-week intervention in PD. Its short and long-term benefits will be evaluated. By using iTBS before VBT we expect to further strengthen the VBT effects, also to achieve sustainable long-term effects. The improved dexterity in both the short and long term, will lead to improved ADL and QoL in patients with PD.

## Data Availability Statement

The original contributions presented in the study are included in the article/supplementary material, further inquiries can be directed to the corresponding author/s.

## Ethics Statement

The studies involving human participants were reviewed and approved by Ethics Committee for Northwest and Central Switzerland (EKNZ), Switzerland 2019-00433. The patients/participants provided their written informed consent to participate in this study.

## Author Contributions

MP-W responsible for patient recruitment, provides data collection, and manuscript writing. DL gave critical review of concept, design of the study, and critically revised the manuscript. TN gave critical review of concept, design of the study, and critically revised the manuscript. SB provided concept and design of the study and critically revised the manuscript conceptualization. TV obtained funding, conceived the idea for the present study, overall project coordination, and manuscript writing. All authors contributed to the design of the interventions and outcome measures. All authors assisted in editing and reviewing the submitted manuscript. They all read and approved the final form.

## Funding

The study is funded by the Jacques and Gloria Gossweiler Foundation.

## Conflict of Interest

The authors declare that the research was conducted in the absence of any commercial or financial relationships that could be construed as a potential conflict of interest.

## Publisher's Note

All claims expressed in this article are solely those of the authors and do not necessarily represent those of their affiliated organizations, or those of the publisher, the editors and the reviewers. Any product that may be evaluated in this article, or claim that may be made by its manufacturer, is not guaranteed or endorsed by the publisher.

## References

[1] Foki T Vanbellingen T Lungu C Pirker W Bohlhalter S Nyffeler T . Limb-kinetic apraxia affects activities of daily living in Parkinson's disease: a multi-center study. Eur J Neurol. (2016) 23:1–10. 10.1111/ene.13021 27132653 PMC5565263

[2] Vanbellingen T Hofmänner D Kübel S Bohlhalter S. Limb kinetic apraxia is an independent predictor for quality of life in Parkinson's disease. Mov Disord Clin Pract. (2018) 5:156–9. 10.1002/mdc3.12572 30363441 PMC6174495

[3] Vanbellingen T Kersten B Bellion M Temperli P Baronti F Müri R . Impaired finger dexterity in Parkinson's disease is associated with praxis function. Brain Cogn. (2011) 77:48–52. 10.1016/j.bandc.06.003 21775040

[4] Vanbellingen T Nyffeler T Nef T Kwakkel G Bohlhalter S van Wegen EE. Reliability and validity of a new dexterity questionnaire (DextQ-24) in Parkinson's disease. Parkinsonism Relat Disord. (2016) 33:78–83. 10.1016/j.parkreldis.09.015 27663063

[5] Gebhardt A Vanbellingen T Baronti F Kersten B Bohlhalter S. Poor dopaminergic response of impaired dexterity in Parkinson's disease: Bradykinesia or limb kinetic apraxia? Mov Disord. (2008) 23:1701–6. 10.1002/mds.22199 18649388

[6] Mateos-Toset S Cabrera-Martos I Torres-Sánchez I Ortiz-Rubio A González-Jiménez E Valenza M. Effects of a single hand-exercise session on manual dexterity and strength in persons with Parkinson disease: a randomized controlled trial. PM R. (2016) 8:115–22. 10.1016/j.pmrj.06.004 26079867

[7] Vanbellingen T Nyffeler T Nigg J Janssens J Hoppe J Nef T . Home based training for dexterity in Parkinson's disease: a randomized controlled trial. Parkinsonism Relat Disord. 41:92–98. 10.1016/j.parkreldis.05.021 28578819

[8] Nieuwboer A Rochester L Müncks L Swinnen SP. Motor learning in Parkinson's disease: limitations and potential for rehabilitation. Parkinsonism Relat Disord. (2009) 15:S53–8. 10.1016/S1353-8020(09)70781-3 20083008

[9] Garcia-Agundez A Folkerts AK Konrad R Caserman P Tregel T Goosses M . Recent advances in rehabilitation for parkinson's disease with exergames: a systematic review. J Neuroeng Rehabil. (2019) 16:1–17. 10.1186/s12984-019-0492-1 30696453 PMC6352377

[10] Harris DM Rantalainen T Muthalib M Johnson L Teo WP. Exergaming as a viable therapeutic tool to improve static and dynamic balance among older adults and people with idiopathic Parkinson's disease: a systematic review and meta-analysis. Front Aging Neurosci. (2015) 7:167. 10.3389/fnagi.2015.00167 26441634 PMC4561514

[11] Fernández-González P Carratalá-Tejada M Monge-Pereira E Collado-Vázquez S Sánchez-Herrera Baeza P Cuesta-Gómez A . Leap motion controlled video game-based therapy for upper limb rehabilitation in patients with Parkinson's disease: a feasibility study. J Neuroeng Rehabil. (2019) 16:1–10. 10.1186/s12984-019-0593-x 31694653 PMC6836460

[12] Oña ED Balaguer C Cano-de la Cuerda R Collado-Vázquez S Jardón A. Effectiveness of serious games for leap motion on the functionality of the upper limb in parkinson's disease: a feasibility study. Comput Intell Neurosci. (2018) 2018:7148427. 10.1155./2018/7148427 29849550 PMC5925003

[13] Sánchez-Herrera-Baeza P Cano-de-la-Cuerda R Oña-Simbaña ED Palacios-Ceña D Pérez-Corrales J Cuenca-Zaldivar JN . The impact of a novel immersive virtual reality technology associated with serious games in Parkinson's disease patients on upper limb rehabilitation: a mixed methods intervention study. Sensors. (2020) 20:2168. 10.3390/s20082168 32290517 PMC7218715

[14] van Beek J van Wegen E Bohlhalter S Vanbellingen T. Exergaming-Based Dexterity training in persons with Parkinson disease: a pilot feasibility study. J Neurol Phys Ther. (2019) 43:168–74. 10.1097/NPT.0000000000000278 31136450

[15] El Tamawy MS Darwish MH El Gohary AM Khalifa HA. S187 Influence of repetitive transcranial magnetic stimulation on cognitive and motor performance in Parkinson's disease patients. Clin Neurophysiol. (2017) 128:e238. 10.1016/j.clinph.07.197

[16] Nardone R Versace V Brigo F Golaszewski S Carnicelli L Saltuari L . Transcranial magnetic stimulation and gait disturbances in Parkinson's disease: a systematic review. Clin Neurophysiol. (2020) 50:213–25. 10.1016/j.neucli.05.002 32620273

[17] Yang C Guo Z Peng H Xing G Chen H McClure M . Repetitive transcranial magnetic stimulation therapy for motor recovery in Parkinson's disease: a meta-analysis. Brain Behav. (2018) 8:e01132. 10.1002/brb3.1132 30264518 PMC6236247

[18] Yang YR Tseng CY Chiou SY Liao KK Cheng SJ Lai KL . Combination of rTMS and treadmill training modulates corticomotor inhibition and improves walking in parkinson disease: a randomized trial. Neurorehabil Neural Repair. (2013) 27:79–86. 10.1177/1545968312451915 22785003

[19] Butler AJ Wolf SL. Putting the brain on the map: use of transcranial magnetic stimulation to assess and induce cortical plasticity of upper-extremity movement. Phys Ther. (2007) 87:719–36. 10.2522/ptj.20060274 17429003

[20] Petzinger GM Fisher BE Van Leeuwen JE Vukovic M Akopian G Meshul CK . Enhancing neuroplasticity in the basal ganglia: the role of exercise in Parkinson's disease. Mov Disord. (2010) 25:S141–5. 10.1002/mds.22782 20187247 PMC4111643

[21] Dimyan MA Cohen LG. Contribution of transcranial magnetic stimulation to the understanding of functional recovery mechanisms after stroke. Neurorehabil Neural Repair. (2010) 24:125–35. 10.1177/1545968309345270 19767591 PMC2945387

[22] Chen YJ Huang YZ Chen CY Chen CL Chen HC Wu CY . Intermittent theta burst stimulation enhances upper limb motor function in patients with chronic stroke: a pilot randomized controlled trial. BMC Neurol. (2019) 19:1–10. 10.1186/s12883-019-1302-x 31023258 PMC6485156

[23] Chung SW Hill AT Rogasch NC Hoy KE Fitzgerald PB. Use of theta-burst stimulation in changing excitability of motor cortex: a systematic review and meta-analysis. Neurosci Biobehav Rev. (2016) 63:43–64. 10.1016/j.neubiorev.01.008 26850210

[24] Ji GJ Liu T Li Y Liu P Sun J Chen X . Structural correlates underlying accelerated magnetic stimulation in Parkinson's disease. Hum Brain Mapp. (2021) 42:1670–81. 10.1002/hbm.25319 33314545 PMC7978118

[25] Di Lazzaro V Pilato F Dileone M Profice P Oliviero A Mazzone P . The physiological basis of the effects of intermittent theta burst stimulation of the human motor cortex. J Physiol. (2008) 586:3871–9. 10.1113/jphysiol.2008.152736 18566003 PMC2538925

[26] Goldsworthy MR Pitcher JB Ridding MC. The application of spaced theta burst protocols induces long-lasting neuroplastic changes in the human motor cortex. Eur J Neurosci. (2012) 35:125–34. 10.1111/j.1460-9568.2011.07924.x 22118241

[27] Huang YZ Rothwell JC Chen RS Lu CS Chuang WL. The theoretical model of theta burst form of repetitive transcranial magnetic stimulation. Clin Neurophysiol. (2011) 122:1011–8. 10.1016/j.clinph.08.016 20869307 PMC3046904

[28] Degardin A Devos D Defebvre L Destée A Plomhause L Derambure P . Effect of intermittent theta-burst stimulation on akinesia and sensorimotor integration in patients with Parkinson's disease. Eur J Neurosci. (2012) 36:2669–78. 10.1111/j.1460-9568.2012.08158.x 22693966

[29] Benninger DH Berman BD Houdayer E Pal N Luckenbaugh DA Schneider L . Intermittent theta-burst transcranial magnetic stimulation for treatment of Parkinson disease. Neurology. (2011) 76:601–9. 10.1212/WNL.0b013e31820ce6bb 21321333 PMC3053339

[30] Shirota Y Ohtsu H Hamada M Enomoto H Ugawa Y. Supplementary motor area stimulation for Parkinson disease: a randomized controlled study. Neurology. (2013) 80:1400–5. 10.1212/WNL.0b013e31828c2f66 23516319

[31] Johns P. Parkinson's disease. In: Clinical Neuroscience. Churchill Livingstone (2014). p. 163–79. 10.1016./B978-0-443-10321-6.00013-8

[32] Nachev P Kennard C Husain M. Functional role of the supplementary and pre-supplementary motor areas. Nat Rev Neurosci. (2008) 9:856–69. 10.1038/nrn2478 18843271

[33] Jenkins IH Fernandez W Playford ED Lees AJ Frackowiak RSJ Passingham RE . Impaired activation of the supplementary motor area in Parkinson's disease is reversed when akinesia is treated with apomorphine. Ann Neurol. (1992) 32:749–57. 10.1002/ana.410320608 1471865

[34] Kübel S Stegmayer K Vanbellingen T Walther S Bohlhalter S. Deficient supplementary motor area at rest: Neural basis of limb kinetic deficits in Parkinson's disease. Hum Brain Mapp. (2018) 39:3691–700. 10.1002/hbm.24204 29722099 PMC6866284

[35] Randhawa BK Farley BG Boyd LA. Repetitive transcranial magnetic stimulation improves handwriting in parkinson's disease. Parkinson's Dis. (2013) 2013:751925. 10.1155./2013/751925 23841021 PMC3681307

[36] Goetz CG Poewe W Rascol O Sampaio C Stebbins GT Counsell C . Movement disorder society task force report on the hoehn and yahr staging scale: status and recommendations. Mov Disord. (2004) 19:1020–8. 10.1002/mds.20213 15372591

[37] Hughes AJ Daniel SE Kilford L Lees AJ. Accuracy of clinical diagnosis of idiopathic Parkinson's disease: a clinico-pathological study of 100 cases. J Neurol Neurosurg Psychiatry. (1992) 55:181. 10.1136/jnnp.55.3.181 1564476 PMC1014720

[38] Gill DJ Freshman A Blender JA Ravina B. The Montreal cognitive assessment as a screening tool for cognitive impairment in Parkinson's disease. Movement Disorders. (2008) 23:1043–6. 10.1002/mds.22017 18381646

[39] Oldfield RC. The assessment and analysis of handedness: The Edinburgh inventory. Neuropsychologia. (1971) 9:97–113. 10.1016/0028-3932(71)90067-4 5146491

[40] Nasreddine ZS Phillips NA Bédirian V Charbonneau S Whitehead V Collin I . The montreal cognitive assessment, MoCA: a brief screening tool for mild cognitive impairment. J Am Geriatr Soc. (2005) 53:695–9. 10.1111/j.1532-5415.2005.53221.x 15817019

[41] Goetz CG Tilley BC Shaftman SR Stebbins GT Fahn S Martinez-Martin P . Movement disorder society-sponsored revision of the unified parkinson's disease rating scale (MDS-UPDRS): scale presentation and clinimetric testing results. Mov Disord. (2008) 23:41–7. 10.1002/mds.22340 19025984

[42] Earhart GM Cavanaugh JT Ellis T Ford MP Foreman KB Dibble L. The 9-hole peg test of upper extremity function: average values, test-retest reliability, and factors contributing to performance in people with parkinson disease. J Neurol Phys Ther. (2011) 35:157–63. 10.1097/NPT.0b013e318235da08 22020457

[43] Proud E Morris ME Bilney B Miller KJ Nijkrake MJ Munneke M . Hand dexterity assessment in Parkinson's disease: construct validity of the 9-hole peg test for the more affected hand. Disabil Rehabil. (2020) 1–5. 10.1080./09638288.2020.1754474 32343614

[44] Mendoza JE Apostolos GT Humphreys JD Hanna-Pladdy B O'Bryant SE. Coin rotation task (CRT): a new test of motor dexterity. Arch Clin Neuropsychol. (2009) 24:287–92. 10.1093/arclin/acp030 19592523

[45] Ineichen-Furrer K Vanbellingen T. Screening für die fingergeschicklichkeit – coin rotation test. Physiopraxis. (2016) 14:48–9. 10.1055/s-0042-10240626606158

[46] Peto V Jenkinson C Fitzpatrick RAY. Determining minimally important differences for the PDQ-39 Parkinson's disease questionnaire. Age Ageing. (2001) 30:299–302. 10.1093/ageing/30.4.299 11509307

[47] Matsunaga K Maruyama A Fujiwara T Nakanishi R Tsuji S Rothwell JC. Increased corticospinal excitability after 5 Hz rTMS over the human supplementary motor area. J Physiol. (2005) 562:295–306. 10.1113/jphysiol.2004.070755 15513947 PMC1665472

[48] Mace M Rinne P Liardon JL Bentley P Burdet E. Comparison of flexible and rigid hand-grip control during a feed-forward visual tracking task. In: IEEE International Conference on Rehabilitation Robotics. IEEE (2015) p. 792–7. 10.1109./ICORR.2015.728129927295638

[49] Johansson RS Westling G. Roles of glabrous skin receptors and sensorimotor memory in automatic control of precision grip when lifting rougher or more slippery objects. Exp Brain Res. (1984) 56:550–64. 10.1007/BF00237997 6499981

[50] Thonnard JL Opsomer L Lefèvre P Pletser V McIntyre J. GRIP: dexterous manipulation of objects in weightlessness. In: Preparation of Space Experiments. (2020). 10.5772./intechopen.93462

[51] Smeragliuolo AH Hill NJ Disla L Putrino D. Validation of the leap motion controller using markered motion capture technology. J Biomech. (2016) 49:1742–50. 10.1016/j.jbiomech.04.006 27102160

[52] Weichert F Bachmann D Rudak B Fisseler D. Analysis of the accuracy and robustness of the leap motion controller. Sensors. (2013) 13:6380–93. 10.3390/s130506380 23673678 PMC3690061

[53] Ahmed MA Darwish ES Khedr EM El Serogy YM Ali AM. Effects of low versus high frequencies of repetitive transcranial magnetic stimulation on cognitive function and cortical excitability in Alzheimer's dementia. J Neurol. (2012) 259:83–92. 10.1007/s00415-011-6128-4 21671144

[54] Chang WH Kim YH Bang OY Kim ST Park YH Lee PKW. Long-term effects of RTMS on motor recovery in patients after subacute stroke. J Rehabil Med. (2010) 42:758–64. 10.2340/16501977-0590 20809058

